# Coffee consumption decreases the connectivity of the posterior Default Mode Network (DMN) at rest

**DOI:** 10.3389/fnbeh.2023.1176382

**Published:** 2023-06-28

**Authors:** Maria Picó-Pérez, Ricardo Magalhães, Madalena Esteves, Rita Vieira, Teresa C. Castanho, Liliana Amorim, Mafalda Sousa, Ana Coelho, Pedro S. Moreira, Rodrigo A. Cunha, Nuno Sousa

**Affiliations:** ^1^Life and Health Sciences Research Institute (ICVS), School of Medicine, University of Minho, Campus de Gualtar, Braga, Portugal; ^2^ICVS/3B's - PT Government Associate Laboratory, Braga/Guimaraes, Portugal; ^3^Clinical Academic Center – Braga, Braga, Portugal; ^4^Departamento de Psicología Básica, Clínica y Psicobiología, Universitat Jaume I, Castellón de la Plana, Spain; ^5^P5 Medical Center, Braga, Portugal; ^6^Psychological Neuroscience Lab, CIPsi, School of Psychology, University of Minho, Braga, Portugal; ^7^CNC-Center for Neuroscience and Cell Biology, University of Coimbra, Coimbra, Portugal

**Keywords:** coffee, resting-state, connectomics, default mode network, executive control network

## Abstract

Habitual coffee consumers justify their life choices by arguing that they become more alert and increase motor and cognitive performance and efficiency; however, these subjective impressions still do not have a neurobiological correlation. Using functional connectivity approaches to study resting-state fMRI data in a group of habitual coffee drinkers, we herein show that coffee consumption decreased connectivity of the posterior default mode network (DMN) and between the somatosensory/motor networks and the prefrontal cortex, while the connectivity in nodes of the higher visual and the right executive control network (RECN) is increased after drinking coffee; data also show that caffeine intake only replicated the impact of coffee on the posterior DMN, thus disentangling the neurochemical effects of caffeine from the experience of having a coffee.

## 1. Introduction

There is a common expectation, namely among habitual coffee drinkers, that coffee increases alertness and psychomotor functioning. For these reasons, many individuals keep drinking coffee to counteract fatigue, stay alert, increase cognitive performance, and increase work efficiency (Smith, 2002). Coffee beverages are constituted of numerous compounds known to affect human behavior, among which are caffeine and chlorogenic acids (Sadiq Butt et al., 2011). From the neurobiological perspective, both caffeine and chlorogenic acids have well-documented psychoactive actions, whereas caffeine is mostly an antagonist of the main adenosine receptors in the brain—A_1_ and A_2A_ receptors, leading to the disinhibition of excitatory neurotransmitter release and enhancement of dopamine transmission via D2 receptors (Fredholm et al., 2005) to sharpen brain metabolism and bolster memory performance (Paiva et al., 2022); chlorogenic acids can directly impact neuronal performance through mechanisms that still need to be understood (Lorist and Tops, 2003; Fernandes et al., 2021).

While the neurochemical action of these compounds seems to be reasonably understood, the psychological effect of coffee/caffeine, although largely genuine, remains a matter of debate and should be considered in the context of its use. In fact, some studies show that caffeine has effects on cognitive performance and mood if taken by non-habitual coffee drinkers, but that these effects may decline due to the development of tolerance (James and Rogers, 2005). While some studies seem to point to an effect of caffeine in decreasing lethargy/fatigue and increasing vigor (Judelson et al., 2013), others suggest that the cognitive and emotional effects only occur after 8 h of abstinence (Heatherley et al., 2005), and others even point to the fact that a significant component of the psychological effect of coffee/caffeine should be attributable to the reversal of adverse withdrawal effects associated with short periods of abstinence from the intake (James and Rogers, 2005) or even to the suggestion of having had coffee beverages (Liguori and Hughes, 1997; Dawkins et al., 2011).

Only limited information is available about the impact of coffee intake on the whole-brain network activity, for which the use of novel imaging techniques has proven to be of relevance. Functional magnetic resonance imaging (fMRI) allows us to study, in a non-invasive way, the function of the human brain during the execution of specific tasks or at rest (Soares et al., 2016). In the context of coffee consumption, some previous studies show that there is the activation of different cortical and subcortical areas during a visuomotor task (Park et al., 2014), or upon the hedonic evaluation of caffeine and saccharin (Gramling et al., 2019), or of the frontopolar and cingulate cortex during a two-back verbal working memory task (Koppelstaetter et al., 2008; Haller et al., 2017), or in a modified Sternberg task (Klaassen et al., 2013). Moreover, recent studies suggest an impact of coffee on brain functional connectivity at rest prompting a functional reorganization toward more efficient network properties with implications in emotionality, alertness, and action readiness (Kim et al., 2021; Magalhães et al., 2021).

Despite these results, there is still an open question on what drives habitual coffee drinkers to keep drinking coffee. Herein, we aim to explore the resting-state fMRI data in a group of habitual coffee drinkers before and after acute coffee consumption, using network functional connectivity approaches. We hypothesize that coffee consumption will lead to higher integration of networks linked to the prefrontal cortex associated with executive memory and with brain health throughout the lifespan, such as the posterior default mode network (DMN) (Buckner et al., 2009; Leech et al., 2011).

## 2. Materials and methods

### 2.1. Ethics statement

The present study was conducted in accordance with the principles expressed in the Declaration of Helsinki (59th amendment) and was approved by the ethics committee of Hospital de Braga. All participants gave written informed consent upon the explanation of the aims of the study and were allowed to withdraw from it and stop their participation in the experiments at any time during the study.

### 2.2. Subject recruitment and assessment

All subjects were recruited from the general healthy Portuguese population. Exclusion criteria included the presence of neurological or psychiatric disorders, habitual consumption of mind-altering substances (such as cannabinoid substances), and the inability to undergo an MRI scanning session. The inclusion criteria in order to be considered a coffee drinker was to drink a minimum of one cup of coffee per day. Then, volunteer subjects who agreed to take part in the study were asked to abstain from consuming caffeinated beverages or food for at least 3 h before participating in the study. In total, 47 subjects were recruited who fulfilled the inclusion criteria. Subjects' sociodemographic data were collected during an interview and then they performed an MRI/fMRI scanning session.

### 2.3. MRI brain imaging

MRI scans were conducted using a Siemens Verio 3T (Siemens, Erlangen, Germany) located in Hospital de Braga (Braga, Portugal) using a 32-channel head antenna. The scanning session included as an anatomical acquisition one sagittal magnetization-prepared rapid acquisition with gradient echo (MPRAGE, TR/TE = 2,420/4.12 ms, FA = 9°, 1 mm^3^ isometric voxel size, and Field-of-View = 176 × 256 × 256 mm^3^). The resting state fMRI (rs-fMRI) acquisition used a multi-band echo planar imaging sequence, CMRR EPI 2D [R2016A, Center for Magnetic Resonance Research, University of Minnesota, Minnesota, USA (Feinberg et al., 2010; Moeller et al., 2010; Xu et al., 2013)] sensitive to fluctuations in the blood oxygenation-dependent level contrast (two sets of parameters were used: TR/TE = 1,190/34 ms, FA = 62°, 2 mm^3^ isometric voxel size, and 76 axial slices over a matrix of 256 × 256 mm^2^; and TR/TE =1,000/27 ms, FA = 62°, 2 mm^3^ isometric voxel size, and 64 axial slices over a matrix of 200 × 200 mm^2^). Each rs-fMRI acquisition had a duration of 7 and a half min, during which subjects were instructed to remain with their eyes closed, relaxed, and let their minds wander freely. Each subject was scanned twice using such acquisitions: a baseline acquisition at the start of the scanning session, immediately followed by coffee intake (containing 85 mg of caffeine over 100 ml of water, no added sugar), and 30 min after coffee intake, a second fMRI acquisition was performed.

### 2.4. Preprocessing of MRI data

MRI results included in this article come from preprocessing performed using fMRIPrep 1.4.1 (Esteban et al., 2019). A full description of the preprocessing pipeline can be found in the Supplementary material.

### 2.5. Resting-state analysis

For all resting-state analyses, a dummy variable was included as a covariate to control for the potential impact of the two different resting-state sequences used. Moreover, sex, age, and education level were also included as covariates. Then, a general-linear model (GLM) was performed in order to remove the effect of the confounding variables from both resting-state acquisitions, and the residuals were used as a signal of interest for the analysis.

#### 2.5.1. Independent component analysis

Resting-state network (RSN) maps were analyzed voxel-wise through a probabilistic independent component analysis (ICA) as implemented in multivariate exploratory linear optimized decomposition into independent components (MELODIC), distributed with FSL (Beckmann and Smith, 2004). Probabilistic ICA is a fully data-driven approach that enables the isolation of components based on the temporal correlation of the corresponding areas while maximizing the spatial independence between components. Then, dual-regression analysis was performed to estimate the subject-specific components that correspond to the group-wise RSNs. Because the probabilistic ICA approach may identify noisy components corresponding to non-biological signals, such as movement artifacts, the independent components were selected after the visual inspection of their spatial distribution (Horowitz-Kraus et al., 2015). Specifically, components that were mainly present in regions that do not generate the blood oxygen level-dependent (BOLD) signal (white matter, ventricles, or outside the brain) were excluded from the analysis.

Then, the RSNs functional connectivity (FC) was compared between both resting-state sequences (after the removal of confounding effects), using paired *t*-tests within a non-parametric permutation procedure implemented in the randomize tool from FSL (Winkler et al., 2014). Threshold-free cluster enhancement (TFCE) was used to detect widespread significant differences and control the family-wise error rate (FWE-R) at α = 0.05. For each contrast, 5,000 permutations were performed.

#### 2.5.2. Functional connectomics analysis

For the functional connectomics analysis, data were first extracted for each subject from a 284 regions atlas that combined 200 cortical regions from the Schaefer parcellation (doi: 10.1093/cercor/bhx179) with 84 subcortical functional regions from the Shen atlas (Shen et al., 2013), thus combining two different functional atlases for full brain coverage. For each subject pair of acquisitions, and to transform the data into matrices of FC, a *Pearson's* correlation between time series was calculated, followed by the Fisher's *Z* transformation to normalize its distribution.

To overcome the issue of multiple comparisons induced by the large number of connections in the network, we applied the network-based statistics (NBS) approach (Zalesky et al., 2010), as we have previously described (Magalhães et al., 2021). This approach provides a correction equivalent to the FWE-R by estimating the probability of identifying, in a random permutation of the tested data, networks to a larger extent than the ones identified in the hypothesis tested. This is done in two phases. The first step tests the statistical hypothesis at each point of the matrix, which is then filtered by a user-chosen statistical edge threshold. Significant network components are identified as sets of threshold-surviving connections between nodes, such that each node in the network can be reached from any other, through significant connections. The component size is calculated as the number of significant connections. While the connection threshold is not directly determinant of the network significance, it determines the possible extent of the network, with lower significance edge thresholds revealing larger and more widespread networks and higher thresholds resulting in smaller and more focused effects. As such, following the toolbox authors' recommendation, we explored a range of edge thresholds between 0.005 and 0.00005. Then, step 2 creates random permutations of the data set and applies the same methodology as step 1 to each permutation, determining the size of network components found. Finally, the calculated distribution of components' size is used to estimate the probability of finding random components with a size greater than the one found in our hypothesis. A total of 5,000 permutations were used, together with a FWE corrected network significance of 0.05. To test the acute impact of caffeine intake, we used a paired *t*-test comparison between the pre- and post-coffee intake acquisitions.

### 2.6. Impact of caffeine on the results

To help understand if the results from the previously described analysis extend beyond the impact of caffeine consumption, we conducted a complementary analysis using a second population of habitual coffee drinkers (*N* = 36). Instead of a coffee drink, subjects from this group were given the same amount of hot water with the same amount of caffeine diluted in it. Following each of the previous analyses, we extracted the data from the networks of interest for the ICA analysis and from the significant network component for NBS and tested for the same effect within the caffeine drinkers. This analysis was conducted using paired sample *t*-tests.

## 3. Results

For the analysis of coffee consumption, we have studied 47 individuals (31 women), with an average age of 30 years (SD 7.9). To discriminate the effect of caffeine in the results, we studied an additional group of 36 individuals (27 women), with 32.1 (SD 11.1) years of age.

### 3.1. Independent-components analysis

**Figure 1:**
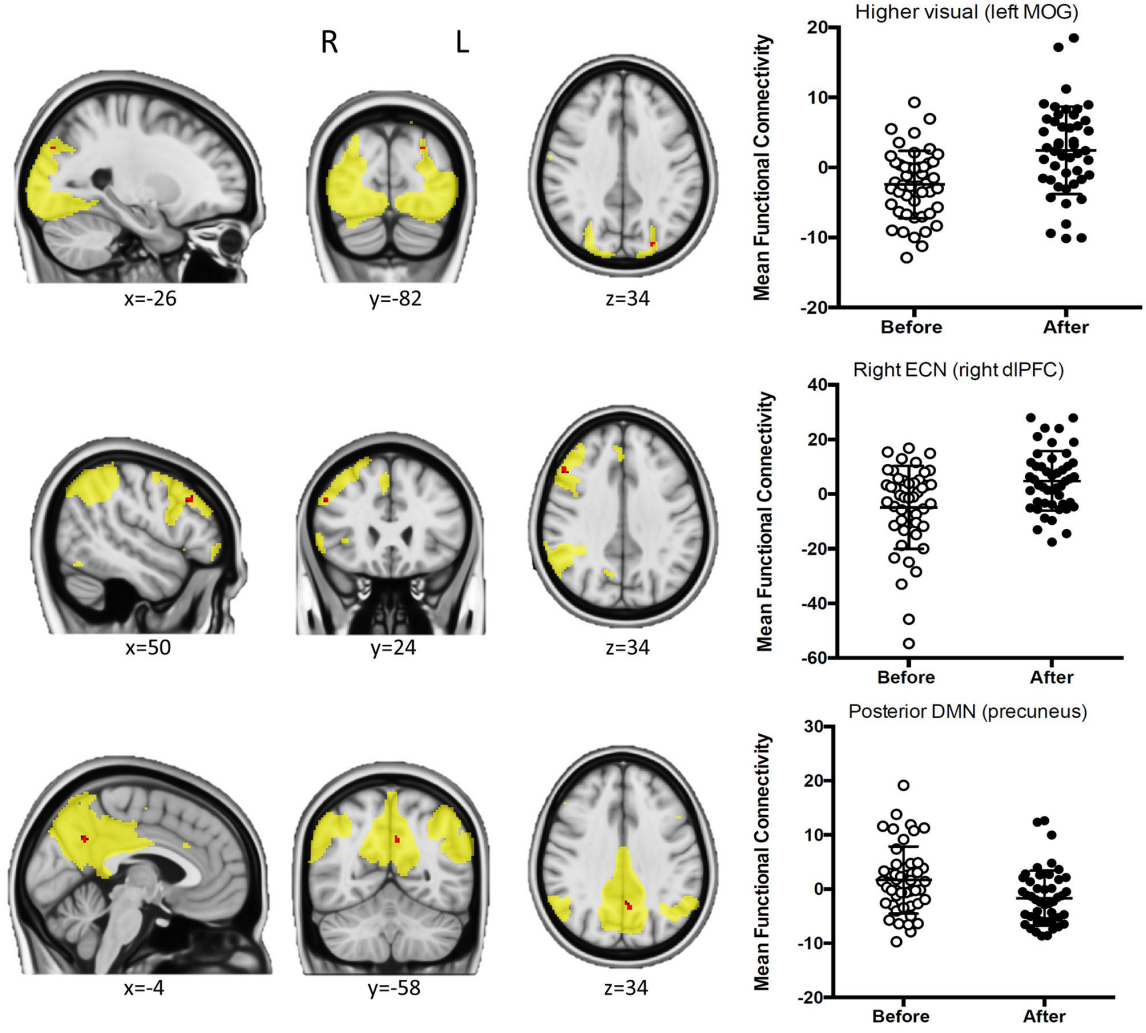
Results from ICA. For those clusters showing significant pre-post coffee intake connectivity differences, sagittal, coronal, and axial views **(left)** of the clusters, and box plots **(right)** representing the mean connectivity before and after are shown. The FWE-R TFCE corrected clusters are presented in red and overlaid on their corresponding networks in yellow.

A total of 30 components were obtained from the probabilistic ICA, and 12 of these components were found to be representative of the most typical RSNs (see Supplementary Figure 1). We found significant FWE-R TFCE corrected pre-post coffee intake differences in several networks (see Figure 1).

In the higher visual network, the connectivity with the left middle occipital gyrus (MOG) was increased after drinking coffee (MNI coordinates = −26, −82, 34; 3 voxels; peak *t*-value = 4.54), as well as the connectivity of the right dorsolateral prefrontal cortex (dlPFC) within the right executive control network (RECN; MNI coordinates = 50, 24, and 34; 13 voxels; peak *t*-value = 4.81).

On the other hand, for the posterior DMN, the connectivity in a cluster within the left precuneus decreased after coffee intake (MNI coordinates = −4, −58, 34; 14 voxels; peak *t*-value = 4.63).

When running paired *t*-tests in the caffeine group for the ICA networks showing significant differences in this analysis (higher visual, RECN, and posterior DMN), we found no significant modulation of the higher visual and the RECN networks, while for the posterior DMN, we found a significant effect and in the same direction than in the group that drank coffee (decreased connectivity after caffeine intake; MNI coordinates = −8, −84, 44; 29 voxels; peak *t*-value = 5.35).

### 3.2. Connectomics analysis

**Figure 2:**
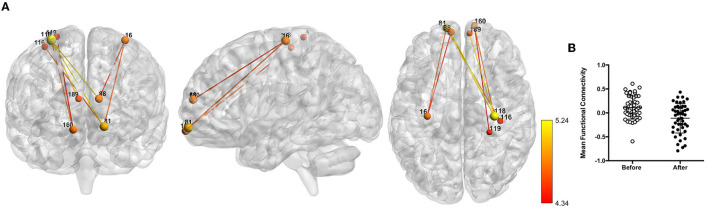
Network of reduced functional connectivity (FC) after coffee consumption for a threshold of *p* < 5e-05. **(A)** Coronal, sagittal, and axial view of the networks, with connections color-coded from red to yellow according to the statistical strength of the effect. **(B)** Box plots representing the before and after coffee consumption average FC.

The NBS analysis revealed a network of decreased functional connectivity that was modulated by coffee consumption. Components with different extents of this network were found to be significant for thresholds higher than 0.001 (statistics for all tested thresholds are presented in Table 1). To facilitate visualization, we present here only the network obtained for a threshold of *p* = 5e-05 [*t*(threshold) = 4.26, network *p* = 0.022, hedge's *g* = 0.71, eight nodes and eight edges; Figure 2]. This network was mostly composed of nodes from the somatosensory/motor and default mode networks and from the prefrontal cortex. Specifically, we highlight regions overlapping the precentral gyrus (nodes #118 and #16), the superior and medial frontal gyrus (#81, #88, #160, and #189), and the middle frontal gyrus (#88). Furthermore, at lower threshold levels, effects can also be found in other brain regions (Supplementary Figure 2).

**Table 1:** Results of the functional connectomics analysis using NBS.

**Threshold (*p*, *t*)**	***p* (network)**	***g***	***N* nodes**	***N* edges**	**FC before**	**FC after**
0.005, 2.69	0.061	–	–	–	–	–
0.001, 3.27	0.036	0.60	86	143	0.087	−0.088
0.0005, 3.52	0.030	0.63	60	81	0.092	−0.091
0.0001, 4.04	0.039	0.70	9	10	0.109	−0.109
0.00005, 4.26	0.022	0.71	8	8	0.112	−0.112

For each of the five thresholds used, we report here the p significance of the networks (for p < 0.1), Hedge's g, as a measure representative of the effect size, as well as the network size represented as the number of nodes, the number of edges in the network, and the mean network FC for each moment.

Testing for significant differences in the caffeine group within the networks obtained in this analysis, we found a trend for a difference in this network (*p* = 0.087).

## 4. Discussion

The current study, by comparing resting-state FC before and after coffee consumption in habitual coffee drinkers, reveals that the connectivity of the posterior DMN is decreased after drinking coffee, while the connectivity in nodes of the higher visual and the right executive control network (RECN) is increased after drinking coffee. Importantly, the decrease in the posterior DMN connectivity is replicated by caffeine intake, whereas the alterations observed in the higher visual and the RECN are not. These observations add to our knowledge of the motivation to drink coffee and disentangle the brain connectivity effects that are attributable to caffeine (posterior DMN), from those that are triggered by other dimensions of coffee intake.

One of the major findings of this study relates to the impact of coffee (and caffeine) on the connectivity of the posterior DMN, namely in the left precuneus. The components of the DMN are known as a cognitive and physiological neurobiological framework necessary for the brain to respond to external stimuli (Buckner et al., 2008). In particular, the precuneus is a dynamic area of the brain involved in self-consciousness, memory, and visuospatial imagery (Fletcher et al., 1995; Lou et al., 2004; Cavanna and Trimble, 2006; Andrews-Hanna et al., 2010; Boly et al., 2012), functions commonly reported to be altered after coffee intake. Similarly, coffee and caffeine are well-known to induce wakefulness, and the finding herein reported is noticeable given that the precuneus network is connected to subcortical areas of the reticular activating system implicated in arousal (Tomasi and Volkow, 2011). Additionally, it is worth noting that the precuneus has emerged as a central node of the DMN and perhaps the most connected hub in the cortex (Cavanna, 2007; Buckner et al., 2008; Tomasi and Volkow, 2011; Utevsky et al., 2014; Pereira-Pedro and Bruner, 2016) and, therefore, the observation of decreased connectivity after coffee and caffeine intake at rest in this node points to a higher preparedness to switch from resting to task-context processing after coffee intake (Childs and De Wit, 2006). Curiously, and in accordance, the precuneus has been implicated in the guidance of motor responses but also in higher achievement motivation, namely in the left hemisphere (Childs and De Wit, 2006).

Another relevant finding of this study was the coffee-associated decreased functional connectivity between the somatosensory/motor networks and the prefrontal cortex; more specifically, there was decreased functional connectivity between the precentral gyrus and the superior and medial middle frontal gyrus after coffee intake. Although not statistically significant, there was a relevant trend toward a similar observation after caffeine intake. This result reveals that caffeine consumption in habitual coffee drinkers prepares them for action. Briefly, the initiation and execution of movements comprise a prefrontal area involved in movement planning, a frontal premotor area involved in movement coordination, the primary motor cortex (i.e., precentral gyrus), from which the final movement command emerges, and the primary sensory cortex (i.e., postcentral gyrus), which allows adaptation through sensory feedback. It has been suggested that the connectivity strength between motor regions and the prefrontal cortex seems to be more associated with cognitive control because the extent to which these interactions occur at rest might be an indication of motor performance during subsequent task performance (Langan et al., 2010; Seidler et al., 2015); this would explain the reported gains in psychomotor efficiency experienced after the intake of caffeinated coffee (Smith, 2002).

Interestingly, increased functional connectivity was observed after coffee, but not caffeine, intake in the higher visual and RECN networks. The middle occipital lobe is implicated in visual processing, and the executive control network (a frontoparietal network that includes among others the dorsolateral prefrontal cortex) is engaged in high-level cognitive functions such as working memory, cognitive control, and goal-directed behavior (Seeley et al., 2007; Menon, 2011; Niendam et al., 2012). Our observation that coffee, but not caffeine, impacts the connectivity in these networks is interesting as higher functional connectivity in executive control and visual networks may underlie cognitive control and visual imagery but also because that is attributable to other components of coffee intake besides caffeine. The latter points to an imaginary sensation (in this case visual) that may trigger pleasure as observed for other stimuli like music (Zatorrea and Salimpoor, 2013) that do not seem to rely on the established neurochemical effects of caffeine in adenosine receptors but rather on the sensory experience of having coffee, which is determined by a complex interaction of multiple compounds present in coffee beverages (Poole and Tordoff, 2017; Lang et al., 2020).

One important issue that should be discussed whenever studying the effect of caffeine in brain networks relates to the design and interpretation of resting-state BOLD fMRI studies, as caffeine is a cerebral vasoconstrictor that may cause an increase in the concentration of deoxyhemoglobin and thus a decrease in the BOLD baseline resting signal (Mulderink et al., 2002). Indeed, it was shown that caffeine reduced the measures of resting-state BOLD connectivity in the motor cortex in parallel with decreased baseline cerebral blood flow and spectral energy in the low-frequency BOLD fluctuations (Rack-Gomer et al., 2009), and another study confirmed not only a reduction in whole-brain global perfusion (Haller et al., 2013) after caffeine but also a caffeine-enhanced task-related activation in a local and distributed network that was most pronounced in the bilateral striatum and to a lesser degree in the right middle and inferior frontal gyrus, bilateral insula, left superior and inferior parietal lobule, as well as in the cerebellum bilaterally. In that same study, it was shown that functional connectivity was significantly enhanced in caffeine vs. placebo in a distributed and task-relevant network including the prefrontal cortex, the supplementary motor area, the ventral premotor cortex, and the parietal cortex, as well as the occipital cortex (visual stimuli) and basal ganglia; importantly, the activation strength of the task-relevant-network component correlated with response accuracy for caffeine yet not for placebo, indicating a selective cognitive effect of caffeine (Haller et al., 2013). Another layer of complexity when analyzing the effects of caffeine in resting-state brain networks relates to the individual heterogeneity in the response and in the nature of the effect. In fact, some studies show that functional connectivity decreases due to caffeine vary among subjects, and a correlation analysis between the changes in functional connectivity and regional blood flow suggested that the effect of caffeine on BOLD functional connectivity was predominantly neural (motor/visual cortices) and partly vascular (DMN). However, it was shown that, after caffeine ingestion, the DMN involved more attentional networks, and more extrastriatal areas were integrated into the functional connectivity of the visual cortex, which may be associated with the known pharmacological effect of caffeine in elevating alertness (Wu et al., 2014). In further support of the cognitive effect of caffeine, it is important to also highlight the findings that the thalamus is involved in mediating the interaction of attention and arousal in an attentional task under different levels of arousal (sleep deprivation or caffeine administration) (Portas et al., 1998) that caffeine stabilizes the extent of neuronal activation in repetitive word stem completion, counteracting the general task practice effect (Bendlin et al., 2007), and the modulatory effect of caffeine on brain regions (medial frontopolar and anterior cingulate cortex) that have been associated with attentional and executive functions (Koppelstaetter et al., 2008).

Finally, it is important to highlight some methodological limitations of the present study. One of the limitations is the absence of a non-drinker control sample (to rule out the withdrawal effect) or an alternative group consuming decaf coffee (to rule out the placebo effect of coffee intake); this fact should be taken into account in future studies on this topic. However, as a methodological strength, we have included a group that took caffeine (instead of coffee) to discriminate the effects that should be attributed to caffeine and not to expectations of having coffee, which is of relevance as there are studies showing that both caffeine and expectation of having consumed caffeine improved attention and psychomotor speed (Dawkins et al., 2011). Another limitation of the current study relates to the fact that we have only studied resting-state brain connectivity, and further studies should also address the impact of coffee/caffeine in task-related events, which are certainly more insightful in the establishment of psycho-cognitive effects of coffee intake and in the discrimination of differences in the patterns of responses to caffeine by habitual consumers and habitual non-consumers. The latter may go some way to explaining why some individuals become caffeine consumers, in light of evidence showing that caffeine tended to benefit consumers' mood more while improving performance more in the non-consumers (Haskell et al., 2005).

## Data availability statement

The raw data supporting the conclusions of this article will be made available by the authors, without undue reservation.

## Ethics statement

The studies involving human participants were reviewed and approved by Ethics Committee of Hospital de Braga. The patients/participants provided their written informed consent to participate in this study.

## Author contributions

NS, RM, PM, and AC contributed to the conception and design of the manuscript. RV, TC, LA, and MS organized the database. MP-P and RM performed the statistical analysis and wrote the first draft of the manuscript. NS and RC wrote sections of the manuscript. All authors contributed to the manuscript revision, read, and approved the submitted version.
