# Supplementary material for: Coffee consumption decreases the connectivity of the posterior Default Mode Network (DMN) at rest
Source: Front Behav Neurosci. 2023 Jun 28;17:1176382. doi: 10.3389/fnbeh.2023.1176382 (PMC10336217; doi:10.3389/fnbeh.2023.1176382)
Supplement: Supplementary file 1 [file Data_Sheet_1.docx]

***Supplementary Material***

**Coffee consumption decreases the connectivity of the posterior DMN at rest**

Maria Picó-Pérez, Ricardo Magalhães, Madalena Esteves, Rita Vieira, Teresa C Castanho, Liliana Amorim, Mafalda Sousa, Ana Coelho, Pedro S Moreira, Rodrigo A Cunha, Nuno Sousa^*^

**^*^Correspondence**: Corresponding Author: njcsousa@med.uminho.pt

**1 Methods**

**1.1 Preprocessing of MRI data**

MRI results included in this manuscript come from preprocessing performed using fMRIPrep 1.4.1 (Esteban et al., 2019)(RRID:SCR_016216), which is based on Nipype 1.2.0 (Gorgolewski et al., 2011)(RRID:SCR_002502).

**1.1.1 Anatomical data preprocessing**

The T1-weighted (T1w) image was corrected for intensity non-uniformity (INU) with N4BiasFieldCorrection (Tustison et al., 2010), distributed with ANTs 2.2.0 (Avants et al., 2008)(RRID:SCR_004757), and used as T1w-reference throughout the workflow. The T1w-reference was then skull-stripped with a Nipype implementation of the antsBrainExtraction.sh workflow (from ANTs), using OASIS30ANTs as target template. Brain tissue segmentation of cerebrospinal fluid (CSF), white-matter (WM) and gray-matter (GM) was performed on the brain-extracted T1w using fast (FSL 5.0.9, RRID:SCR_002823 (Zhang et al., 2001)). Brain surfaces were reconstructed using recon-all (FreeSurfer 6.0.1, RRID:SCR_001847 (Dale et al., 1999)), and the brain mask estimated previously was refined with a custom variation of the method to reconcile ANTs-derived and FreeSurfer-derived segmentations of the cortical gray-matter of Mindboggle (RRID:SCR_002438 (Klein et al., 2017)). Volume-based spatial normalization to standard space (ICBM 152 Nonlinear Asymmetrical template version 2009c (Fonov et al., 2009)(RRID:SCR_008796; TemplateFlow ID: MNI152NLin2009cAsym) was performed through nonlinear registration with antsRegistration (ANTs 2.2.0), using brain-extracted versions of both T1w reference and the T1w template.

**1.1.2 Resting-state data preprocessing**

For each subject, the following preprocessing steps were performed: First, a reference volume and its skull-stripped version were generated using a custom methodology of fMRIPrep. A deformation field to correct for susceptibility distortions was estimated based on a field map that was co-registered to the BOLD reference, using a custom workflow of fMRIPrep derived from D. Greve’s epidewarp.fsl script and further improvements of HCP Pipelines (Glasser et al., 2013). Based on the estimated susceptibility distortion, an unwarped BOLD reference was calculated for a more accurate co-registration with the anatomical reference. The BOLD reference was then co-registered to the T1w reference using bbregister (FreeSurfer) which implements boundary-based registration (Greve and Fischl, 2009). Co-registration was configured with nine degrees of freedom to account for distortions remaining in the BOLD reference. Head-motion parameters with respect to the BOLD reference (transformation matrices, and six corresponding rotation and translation parameters) are estimated before any spatiotemporal filtering using mcflirt (FSL 5.0.9, (Jenkinson et al., 2002)). BOLD runs were slice-time corrected using 3dTshift from AFNI 20160207 (Cox, 1996)(RRID:SCR_005927). The BOLD time-series (slice-time corrected) were resampled onto their original, native space by applying a single, composite transform to correct for head-motion and susceptibility distortions, and finally resampled into MNI152NLin2009cAsym space. Several confounding time-series were calculated based on the preprocessed BOLD: framewise displacement (FD), DVARS and three region-wise global signals. FD and DVARS are calculated for each functional run, both using their implementations in Nipype (following the definitions by (Power et al., 2014)). The three global signals are extracted within the CSF, the WM, and the whole-brain masks. Additionally, a set of physiological regressors were extracted to allow for component-based noise correction (CompCor, (Behzadi et al., 2007)). Principal components are estimated after high-pass filtering the preprocessed BOLD time-series (using a discrete cosine filter with 128s cut-off) for the anatomical variant (aCompCor). A mask covering the subcortical regions was obtained by heavily eroding the brain mask, which ensures it does not include cortical GM regions. For aCompCor, components are calculated within the intersection of the aforementioned mask and the union of CSF and WM masks calculated in T1w space, after their projection to the native space of each functional run (using the inverse BOLD-to-T1w transformation). Components are calculated separately within the WM and CSF masks. For each CompCor decomposition, the k components with the largest singular values are retained, such that the retained components’ time series are sufficient to explain 50 percent of variance across the nuisance mask (CSF, WM, combined, or temporal). The remaining components are dropped from consideration. The mean CSF and WM signals, as well as the first 6 aCompCor components, the FD and the DVARS were regressed as confounds from the BOLD data using fslregfilt. Finally, fslmaths was used to spatially smooth (with a FWHM kernel of 6mm) and band-pass filter (between 0.01 and 0.08 Hz) the resulting time-series.

**2 Results**


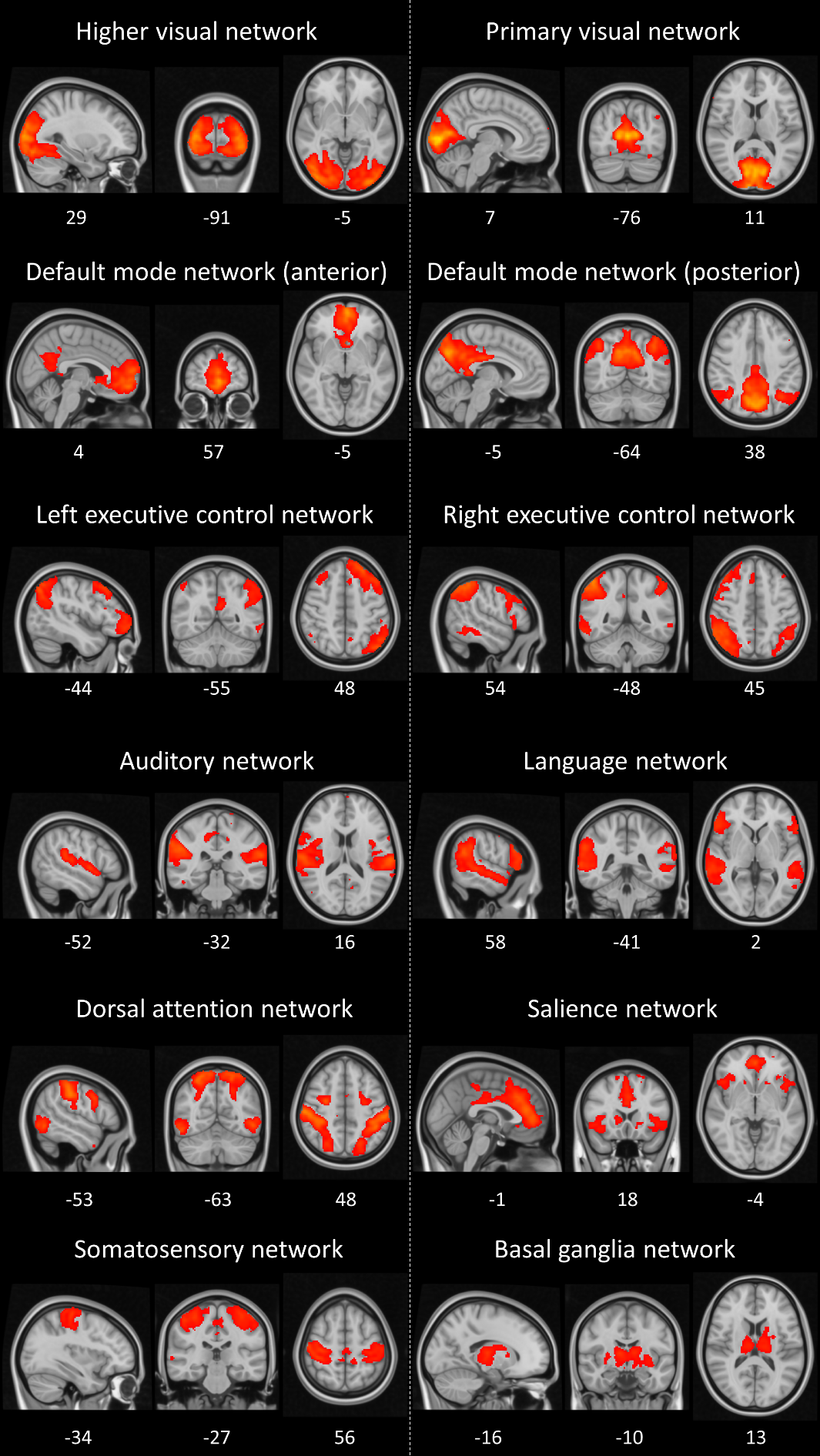


**Supplementary Figure 1.** Resting-state networks identified through probabilistic ICA. Coordinates are x, y, z MNI coordinates.


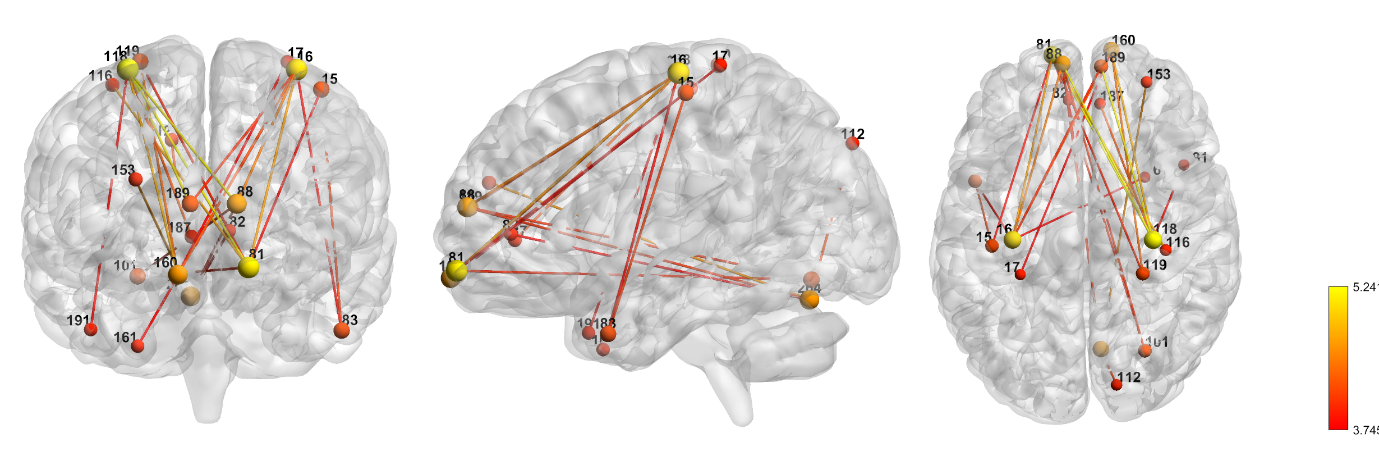
**Supplementary Figure 2.** NBS analysis resulting network, revealing other nodes at a lower threshold.

**References**

Avants, B. B., Epstein, C. L., Grossman, M., and Gee, J. C. (2008). Symmetric diffeomorphic image registration with cross-correlation: Evaluating automated labeling of elderly and neurodegenerative brain. *Med. Image Anal.* 12, 26–41. doi: 10.1016/J.MEDIA.2007.06.004.

Behzadi, Y., Restom, K., Liau, J., and Liu, T. T. (2007). A component based noise correction method (CompCor) for BOLD and perfusion based fMRI. *Neuroimage* 37, 90–101. doi: 10.1016/J.NEUROIMAGE.2007.04.042.

Cox, R. W. (1996). AFNI: software for analysis and visualization of functional magnetic resonance neuroimages. *Comput. Biomed. Res.* 29, 162–173. doi: 10.1006/cbmr.1996.0014.

Dale, A. M., Fischl, B., and Sereno, M. I. (1999). Cortical Surface-Based Analysis: I. Segmentation and Surface Reconstruction. *Neuroimage* 9, 179–194. doi: 10.1006/NIMG.1998.0395.

Esteban, O., Markiewicz, C. J., Blair, R. W., Moodie, C. A., Isik, A. I., Erramuzpe, A., et al. (2019). fMRIPrep: a robust preprocessing pipeline for functional MRI. *Nat. Methods* 16, 111–116. doi: 10.1038/s41592-018-0235-4.

Fonov, V., Evans, A., McKinstry, R., Almli, C., and Collins, D. (2009). Unbiased nonlinear average age-appropriate brain templates from birth to adulthood. *Neuroimage* 47, S102. doi: 10.1016/S1053-8119(09)70884-5.

Glasser, M. F., Sotiropoulos, S. N., Wilson, J. A., Coalson, T. S., Fischl, B., Andersson, J. L., et al. (2013). The minimal preprocessing pipelines for the Human Connectome Project. *Neuroimage* 80, 105–124. doi: 10.1016/J.NEUROIMAGE.2013.04.127.

Gorgolewski, K., Burns, C. D., Madison, C., Clark, D., Halchenko, Y. O., Waskom, M. L., et al. (2011). Nipype: A flexible, lightweight and extensible neuroimaging data processing framework in Python. *Front. Neuroinform.* 5, 13. doi: 10.3389/FNINF.2011.00013/ABSTRACT.

Greve, D. N., and Fischl, B. (2009). Accurate and robust brain image alignment using boundary-based registration. *Neuroimage* 48, 63–72. doi: 10.1016/J.NEUROIMAGE.2009.06.060.

Jenkinson, M., Bannister, P., Brady, M., and Smith, S. (2002). Improved Optimization for the Robust and Accurate Linear Registration and Motion Correction of Brain Images. *Neuroimage* 17, 825–841. doi: 10.1006/NIMG.2002.1132.

Klein, A., Ghosh, S. S., Bao, F. S., Giard, J., Häme, Y., Stavsky, E., et al. (2017). Mindboggling morphometry of human brains. *PLOS Comput. Biol.* 13, e1005350. doi: 10.1371/JOURNAL.PCBI.1005350.

Power, J. D., Mitra, A., Laumann, T. O., Snyder, A. Z., Schlaggar, B. L., and Petersen, S. E. (2014). Methods to detect, characterize, and remove motion artifact in resting state fMRI. *Neuroimage* 84, 320–341. doi: 10.1016/J.NEUROIMAGE.2013.08.048.

Tustison, N. J., Avants, B. B., Cook, P. A., Zheng, Y., Egan, A., Yushkevich, P. A., et al. (2010). N4ITK: Improved N3 bias correction. *IEEE Trans. Med. Imaging* 29, 1310–1320. doi: 10.1109/TMI.2010.2046908.

Zhang, Y., Brady, M., and Smith, S. (2001). Segmentation of brain MR images through a hidden Markov random field model and the expectation-maximization algorithm. *IEEE Trans. Med. Imaging* 20, 45–57. doi: 10.1109/42.906424.
